# Antiviral Potential of a Novel Compound CW-33 against Enterovirus A71 via Inhibition of Viral 2A Protease

**DOI:** 10.3390/v7062764

**Published:** 2015-06-17

**Authors:** Ching-Ying Wang, An-Cheng Huang, Mann-Jen Hour, Su-Hua Huang, Szu-Hao Kung, Chao-Hsien Chen, I-Chieh Chen, Yuan-Shiun Chang, Jin-Cherng Lien, Cheng-Wen Lin

**Affiliations:** 1 Department of Medical Laboratory Science and Biotechnology, China Medical University, Taichung 40402, Taiwan; E-Mails: spirit1126@hotmail.com (C.-Y.W.); chchen@mail.cmu.edu.tw (C.-H.C.); hiichieh@gmail.com (I.-C.C.); 2 School of Chinese Pharmaceutical Sciences and Chinese Medicine Resources, China Medical University, Taichung 40402, Taiwan; E-Mail: yschang@mail.cmu.edu.tw; 3 Department of Nursing, St. Mary’s Junior College of Medicine, Nursing and Management, Yilan County 266, Taiwan; E-Mail: haj@smc.edu.tw; 4 School of Pharmacy, China Medical University, Taichung 40402, Taiwan; E-Mail: mjhou@mail.cmu.edu.tw; 5 Department of Biotechnology, Asia University, Wufeng, Taichung 41354, Taiwan; E-Mail: shhuang@asia.edu.tw; 6 Department of Biotechnology and Laboratory Science in Medicine, National Yang Ming University, Taipei 11221, Taiwan; E-Mail: szkung@ym.edu.tw

**Keywords:** enterovirus A71, 2A protease, type I interferon, antagonism, inhibitor

## Abstract

Enterovirus A71 (EV-A71) in the *Picornaviridae* family causes hand-foot-and-mouth disease, aseptic meningitis, severe central nervous system disease, even death. EV-A71 2A protease cleaves Type I interferon (IFN)-α/β receptor 1 (IFNAR1) to block IFN-induced Jak/STAT signaling. This study investigated anti-EV-A7l activity and synergistic mechanism(s) of a novel furoquinoline alkaloid compound CW-33 alone and in combination with IFN-β. Anti-EV-A71 activities of CW-33 alone and in combination with IFN-β were evaluated by inhibitory assays of virus-induced apoptosis, plaque formation, and virus yield. CW-33 showed antiviral activities with an IC_50_ of near 200 μM in EV-A71 plaque reduction and virus yield inhibition assays. While, anti-EV-A71 activities of CW-33 combined with 100 U/mL IFN-β exhibited a synergistic potency with an IC_50_ of approximate 1 μM in plaque reduction and virus yield inhibition assays. Molecular docking revealed CW-33 binding to EV-A71 2A protease active sites, correlating with an inhibitory effect of CW33 on *in vitro* enzymatic activity of recombinant 2A protease (IC_50_ = 53.1 μM). Western blotting demonstrated CW-33 specifically inhibiting 2A protease-mediated cleavage of IFNAR1. CW-33 also recovered Type I IFN-induced Tyk2 and STAT1 phosphorylation as well as 2′,5′-OAS upregulation in EV-A71 infected cells. The results demonstrated CW-33 inhibiting viral 2A protease activity to reduce Type I IFN antagonism of EV-A71. Therefore, CW-33 combined with a low-dose of Type I IFN could be applied in developing alternative approaches to treat EV-A71 infection.

## 1. Introduction

Enterovirus A71 (EV-A71) belongs to the “Enterovirus A” species, genus *Enterovirus* in the *Picornaviridae* family, comprising an icosahedral capsid and single positive-strand RNA genome of approximately 7400 nucleotides [1]. The genus *Enterovirus*, one of most common genera within the family *Picornaviridae*, comprises 72 serotypes: e.g., poliovirus, Coxsackie A virus (CVA), Coxsackie B virus (CVB), echovirus, EV-A71 [2,3,4]. EV-A71 was first isolated and characterized from cases of neurological disease in California as of 1969 [5], usually infecting children by direct contact with virus shed from the upper respiratory or gastrointestinal tract. EV-A71 causes hand-foot-and-mouth disease (HFMD), aseptic meningitis, exanthems, acute flaccid paralysis, pericarditis, severe central nervous system diseases, even death. The genome contains a single long open reading frame (ORF) and untranslated regions (UTR) at 5′ and 3′ ends. The ORF encodes a polyprotein precursor cleaved into many consecutive functional parts by viral 2A and 3C proteases, such as P1, P2 and P3 fragments firstly cleaved by 2A protease. The P1 fragment yields structural proteins VP1, VP2, VP3 and VP4, while P2 and P3 fragments divide into non-structural 2A, 2B, 2C, 3A, 3B, 3C and 3D.

EV-A71 outbreaks occur worldwide, especially in the Asia-Pacific region. A 1997 epidemic in Malaysia caused 31 fatalities. In Taiwan, it caused 788 deaths in 1998 and 51 in 2001–2002 [1]. The 2010 outbreak in China saw over 1 million cases: 15,000 severe, with over 600 fatalities. EV-A71 remains a global menace, with no effective vaccine for clinical use, making EV71 vaccine a primary candidate for development in the non-clinical stage [6]. Recently, Taiwan’s National Health Research Institutes (NHRI) completed the first Phase I clinical trial of such a vaccine for children [6]. However, specific preventive agents against EV-A71 are not available at present. Interferons (IFNs), effectively antiviral cytokines, are used in combination with antiviral drugs (ribavirin, boceprevir and telaprevir) for hepatitis B or C treatment [7,8]. Because IFNs exhibit lesser potential against EV-A71 [9,10], rare reports indicate them as clinical treatment for EV-A71 infection [11]. Side-effects will often appear—e.g., fever, chills, headache, muscle ache/pain, malaise—after IFN injection.

Pleconaril, a clinical compound targeting VP1, successfully inhibits rhinovirus and some enteroviruses, but not EV-A71 [12]. Rupintrivir is the most successful peptidomimetic 3C^pro^ inhibitor against rhinovirus, CVB2, CVB5, EV-6, and EV-9 in Phase II clinical trials [6,13,14]. A series of BPROZ imidazolidinone derivatives based on “Win” template structures, like WIN 51711 (5-[7-[4-(4,5-dihydro-2-oxazolyl)phenoxy] heptyl]-3-methylisoxazole) are reported to target EV-A71 VP1, inhibiting EV-A71 replication *in vitro* [15]. Lactoferrin, allophycocyanin, and Chinese herbal compounds (eupafolin, ursolic acid, chrysosplenetin, pendulentin, geniposide, and aloe-emodin) display *in vitro* antiviral activity against EV-A71 [16,17]. However, anti-EV-A71 agents are still in development for clinical use.

**Figure 1:**
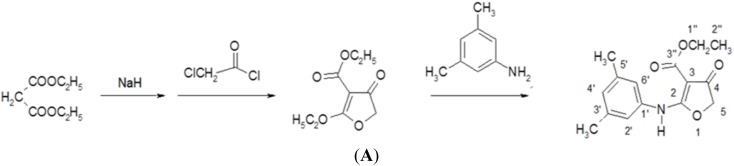

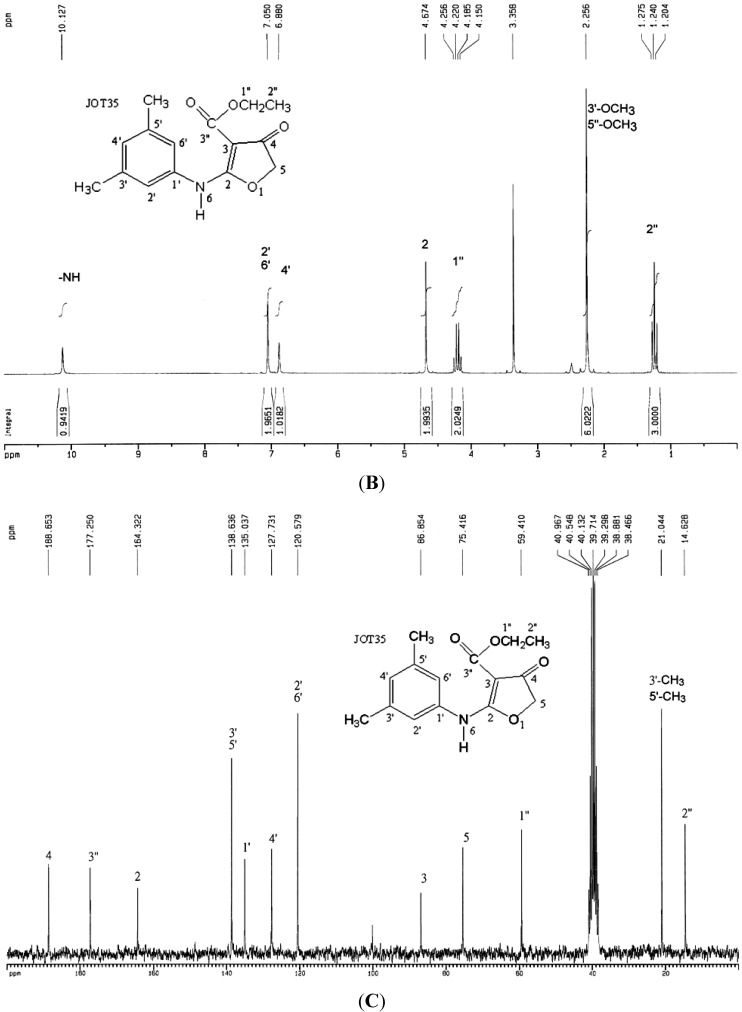
CW-33 chemical synthesis and NMR characterization (Ethyl 2-(3′,5′-dimethylanilino)-4-oxo-4,5-dihydrofuran-3-carboxylate). Flow diagram of CW-33 synthesis appears in (**A**). ^1^H-NMR and ^13^C-NMR spectra of CW-33 are shown in (**B**,**C**), respectively.

Furoquinoline alkaloids are bioactive compounds in many plants in the Rutaceae family, such as Hortia oreadica, H. apiculata, Teclea afzelii, Oricia suaveolens, and Balfourodendron riedelianum [18]. Most furoquinoline alkaloids possess many biological activities: e.g., antifungal [19], antimicrobial [20], antioxidant [21], and anticancer activities [22]. Several synthesized compounds based on furoquinoline skeleton, such as n-alkyl-2,3,4,9-tetrahydrofuro [2,3-b] quinoline-3,4-diones and *N*-benzyl-7-methoxy-2,3,4,9-tetrahydrofuro[2,3-*b*]quinoline-3,4-dione (HA-7), exhibit anti-inflammatory, antiallergic and antiarrhythmic activities [23,24]. Compound CW-33 is a novel synthetic derivative of furoquinoline alkaloid; flow diagram of CW-33 synthesis appears in Figure 1A. This study rates anti-EV-A7l activity of a novel furoquinoline alkaloid compound CW-33 alone and in combination with IFNβ. CW-33 shows an inhibitory effect on EV-A71 replication *in vitro*. Combined treatment with CW-33 and IFN-β exhibits a synergistic antiviral effect on reducing EV-A71-induced cytopathy (apoptosis), virus yield, and plaque formation. Molecular docking analysis indicated the binding of CW-33 to EV-A71 2A protease active sites, confirmed by the inhibitory effect of CW-33 on recombinant 2A enzymatic activity as well as recovering the protein levels of IFNAR1 in EV-A71-infected cells.

## 2. Materials and Methods

### 2.1. Viruses and Cells

EV-A71 strain CMUH2005/V978, isolated from throat swab culture of a young child with encephalitis [25], grew in RD cells. The cells maintained at 37 °C, 5% CO_2_ in Dulbecco’s Modified Eagle’s Medium (DMEM) with 10% fetal bovine serum (FBS). Titers of EV-A71 were quantified by plaque assay on RD cell monolayer, stocks stored at −80 °C until use, as described in prior reports [26,27].

### 2.2. Synthesis of Compound CW-33

Compound CW-33 (ethyl 2-(3′,5′-dimethylanilino)-4-oxo-4,5-dihydrofuran-3-carboxylate was synthesized (Figure 1A). Briefly, 250 mL of 6 M sodium hydride in tetrahydrofuran (THF) was slowly added to 250 mL of 6 M diethyl malonate in THF by shaking for 20 min, then bathed in water at 10–12 °C. Then, 400 mL of 2 M chloroacetyl chloride in THF was added dropwise to the mixture over 1 h, incubated at 40–50 °C for another hour and cooled to 10–12 °C; 3,5-dimethylaniline (0.75 mole) in THF was added dropwise to the reaction solution over 1 h. Finally, reaction mixture left at room temperature overnight was heated under reflux for 2 h, then cooled and poured into ice water. Solid precipitate was extracted with chloroform, washed with water, and dried with magnesium. Solvent was partially evaporated, with concentrated residue refrigerated for six days; precipitate was subsequently collected and recrystallized from ethanol to form compound CW-33 (21.7 g, yield 79%). After purification by high-performance liquid chromatography, CW-33 identity and purity were confirmed by nuclear magnetic resonance (NMR) spectroscopy and mass spectrum. Melting point (m.p.): 144–147 °C; MS (*m/z*): 275 (M^+^); IR (KBr disc, cm^−1^): 3251 (–NH–), 1708 (C4=O), 1662 (C3-CO–OEt); UVλmax nm(CHCl_3_) (log): 283 (4.4); 297 (4.5); ^1^H-NMR (200 MHz, DMSO-d6) (Figure 1B): 1.24 (3H, *t*, *J* = 7.0 Hz, H-2′′), 2.25 (6H, *s*, C3′-CH_3_, C5′-CH_3_), 4.20 (2H, *q*, *J* = 7.0 Hz, H-1′′), 4.67 (2H, *s*, H-5), 6.88 (1H, *s*, H-4′), 7.05 (2H, *s*, H-2′, H-6′), 10.13 (1H, *s*, NH); ^13^C-NMR (200 MHz, DMSO-d6) δ (Figure 1C): 14.62 (C-2′′), 21.04 (3′-CH_3_, 5′-CH_3_), 59.41 (C-1′′), 75.41 (C-5), 86.85 (C-3), 120.58 (C-2′, C-6′), 127.73 (C-4′), 135.03 (C-1′), 135.03 (C-3′, C-5′), 164.32 (C-2), 177.25 (C-3′′), 188.65 (C-4).

### 2.3. Cell Cytotoxicity Assay

RD cells (3 × 10^4^ per well) were added into 96-well plates, incubated at 37 °C, 5% CO_2_ overnight, then treated with or without CW-33 (100, 200, 500, 700, or 1000 μM) and DMSO (5%). Survival rates of mock and treated cells were rated by MTT assay 48 h post-treatment, cells reacted with MTT solution for 4 h; insoluble purple formazan converted from MTT by dehydrogenase enzymes of live cells was dissolved by isopropanol/HCl (300:1 (*v*/*v*)). Survival rate was derived from ratio of optical density (OD)_570-630 nm_ of treated cells to OD_570-630 nm_ of mock cells, as described in prior reports [26,27].

### 2.4. Cytopathic Effect (CPE) Reduction and Virus Yield Assays

RD cells were cultured in 6-well plates (37 °C, 5% CO_2_) overnight and infected with EV-A71 at multiplicity of infection (MOI) of 0.1 and simultaneously treated with or without single and combination of CW-33 (2.5, 25, and 125 μM) and IFN-β (100 or 1000 U/mL) (Hoffmann-La Roche, Basel, Switzerland). Cytopathic effect was photographed by inverted microscope 24 and 48 h post-infection. Supernatant harvested from each well quantified virus yield by plaque assay 48 h post-infection: each serially diluted, then added onto monolayer of RD cells, following overlaying 3% agarose in DMEM with 2% FBS. Cell monolayer was stained with 0.1% Crystal Violet 48 h post- incubation in 37 °C and 5% CO_2_. Plaque number count measured virus yield.

### 2.5. Plaque Reduction Assay for 50% Inhibitory Concentration (IC_50_)

Monolayer of RD cells in each well of 6-well plates was infected with EV-A71 (50 pfu) and simultaneously treated with or without single and combined CW-33 (2.5, 25, 125, or 250 μM) and IFN-β (10, 100, or 1000 U/mL). After 48-h incubation (37 °C, 5% CO_2_), plaque was quantified after staining by 0.1% Crystal Violet and 50% inhibitory concentration (IC_50_) from three independent experiments, as described earlier [26,27].

### 2.6. Cell Cycle Analysis of Flow Cytometry

RD cells (4 × 10^5^) infected with EV-A71 (MOI 0.1) in the presence and absence of CW-33, IFN-β, or combination thereof were cultured for 36 h. Cells were washed in PBS, trypsinized, collected, and centrifuged at 2000 rpm for 3 min, pellets dissolved with 490 μL of binding buffer and 5 μL of propidium ioidide (PI)/Annexin V-FITC reagent (Apoptosis Detection Kit, BioVision). After 10-min incubation at room temperature in the dark, cells were analyzed by flow cytometry (BD FACSAria, Becton Dickinson) with 488 nm excitation and 633 nm emission wavelength.

### 2.7. Molecular Docking

To model CW-33 interaction with viral EV-A71 2A protease, crystal structure of 2A proteinase C110A mutant (PDB: 3w95) deposited in the RCSB Protein Data Bank (Avaliable online: http://www.rcsb.org/pdb) served as template. Mu *et al.* derived crystal structure by X-ray diffraction with resolution of 1.85 Å, revealing active site as composed of catalytic triads C110A, H21 and D39, where acidic member of D39 stabilizes active site geometry by centering hydrogen binding network with H21, N19, Y90, and S125 [28]. Molecular docking used LibDock program within software package Discovery Studio 2.5 (Accelrys, San Diego, CA, USA). First, build mutants protocol was used to exchange alanine residue at position 110 to cysteine in order to return all amino acid residues of 3w95 to original 2A proteinase sequence. Protein site features defined by LibDock were labeled as HotSpots prior to docking. Rigid ligand poses were placed into the active site, HotSpots matched as triplets. In the structure of EV-A71 2A protease, Asn19, His21, Asp39, Tyr 90, Ala110, and Ser125 amino acids were defined as active site (sphere radius: 11.5015 Å) [28]. Poses were pruned, final optimization step performed, and the best scoring poses subsequently reported.

### 2.8. In Vitro Enzymatic Assay of Recombinant 2A Protease

Recombinant EV-A71 2A protease was synthesized in *E. coli*, as detailed in prior study [26]. Briefly, expression vector pET24a containing protease gene was transformed into *E. coli* BL21 (DE3), with 10 mL overnight culture of a single colony injected into 400 mL of fresh LB medium containing 25 µg/mL kanamycin for 3 h, induced with 1 mM IPTG for 4 h, harvested by centrifuge at 6000 rpm for 30 min, then resuspended in denaturing buffer (10 mM imidazole, 8 M urea and 1 mM β-mercaptoethanol) before subjecting to sonication. Recombinant 2A (r2A) protease was purified with Ni-NTA column by gradient elution with 25 mM Tris-HCl, pH 7.5, 150 mM NaCl and 300 mM imidazole. Horseradish peroxidase (10 µg/mL) containing Leu-Gly pairs at residues 122–123 served as substrate, incubated 2 h with or without 5 µg/mL of r2A protease and indicated CW-33 concentrations at 37 °C in 96-well plates *in vitro*. Remaining substrate in each reaction was derived with chromogenic substrate ABTS/H_2_O_2_; intensity of the developed color was gauged at 405 nm. Inhibition of r2A protease enzymatic activity was determined as (OD405_subtrsate+CW-33+r2Ao_ − OD405_subtrsate+r2A_)/(OD405_subtrsate_ − OD405_subtrsate+r2Apro_) × 100%.

### 2.9. Western Blot Analysis

Lysates from un-infected, un-infected/treated, virus-infected/untreated, and virus-infected/treated cells were dissolved in SDS-PAGE sample buffer containing 2-mercaptoethanol, boiled for 10 min, then applied to run 8% SDS-PAGE gels. After electronically transferring to nictrocellulose membranes, blots were blocked with 5% skim milk in TBST, then incubated with specific antibodies including anti-phospho-STAT1, anti-phospho-ERK1/2, anti-phospho-p38 MAPK, anti-phospho-Tyk2, anti-IFNAR1, or anti-β-actin antibodies (Cell Signaling Technology, Danvers, MA, USA), respectively. After reaction with horseradish peroxidase-conjugated secondary antibodies against mouse or rabbit IgG, immunoreactive bands were developed, using enhanced chemiluminescent substrates (Amersham Pharmacia Biotech, Piscataway, NJ, USA).

### 2.10. Real-Time Reverse Transcription-Polymerase Chain Reaction (RT-PCR)

Total RNAs isolated from EV-A71 infected RD cells treated with CW-33 alone or combined with IFN-β, using total RNA purification system (Invitrogen), were reverse-transcribed with oligo dT primer and SuperScript III reverse transcriptase kit (Invitrogen). To analyze gene expression in response to CW-33 alone or combined with IFNβ, quantitative PCR used cDNAs, primer pairs, and SYBR Green I PCR Master Mix. Pairs were forward 5′-GATGTGCTGCCTGCCTTT-3′ and reverse primer 5′-TTGGGGGTTAGGTTTATAGCTG-3′ for human 2′,5′-OAS (2′,5′-oligoadenylate synthetase), forward 5′-CATGGGCTGGGACCTGA CGGTGAAG-3′ and reverse primer 5′-CTGCTGCGGCCCTTGTTATT-3′ for IFNAR1 (interferon-α/β receptor 1), or forward 5′-AGCCACATCGCTCAGACAC-3′ and reverse primer 5′-GCCCCAATACGACCAAATCC-3′ for glyceraldehyde-3-phosphate dehydrogenase (GAPDH). Real-time PCR was completed by amplification protocol consisting of 1 cycle at 50 °C for 2 min, 1 cycle at 95 °C for 10 min, 45 cycles at 95 °C for 15 s, and 60 °C for 1 min. Products were detected in ABI PRISM 7700 sequence detection system (PE Applied Biosystems); relative change in mRNA levels of indicated genes were normalized by mRNA level of housekeeping gene GAPDH.

### 2.11. Statistical Analysis

Data from three independent experiments, representing mean ± standard deviation (S.D.), were statistically analyzed by ANOVA, SPSS program (Version 10.1, SPSS Inc.; Chicago, IL, USA), and Scheffe test, with *p* value < 0.05 as statistically significant.

## 3. Results

### 3.1. Antiviral Activity of CW-33 against EV-A71

**Figure 2:**
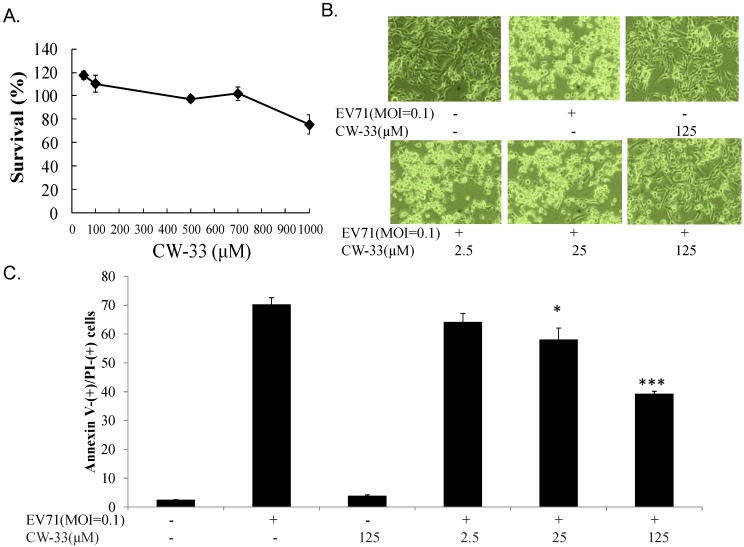
Reducing EV-A71-infected cytopathic effects by CW-33. Firstly, cytotoxicity of CW-33 was performed using MTT assays (**A**). Survival rates of cells were calculated as the ratio of OD_570–630 nm_ of treated cells to OD_570–630 nm_ of untreated cells. Next, antiviral activity of CW-33 was evaluated using the reduction of virus-induced cytopathic effect and apoptosis. Virus-induced cytopathicity was photographed post-infection by phase-contrast microscopy (**B**), while apoptosis of infected and/or treated cells were analyzed by flow cytometry with Annexin-V/PI stain (**C**). * *p* value < 0.05; *** *p* value < 0.001 by Scheffe test.

**Figure 3:**
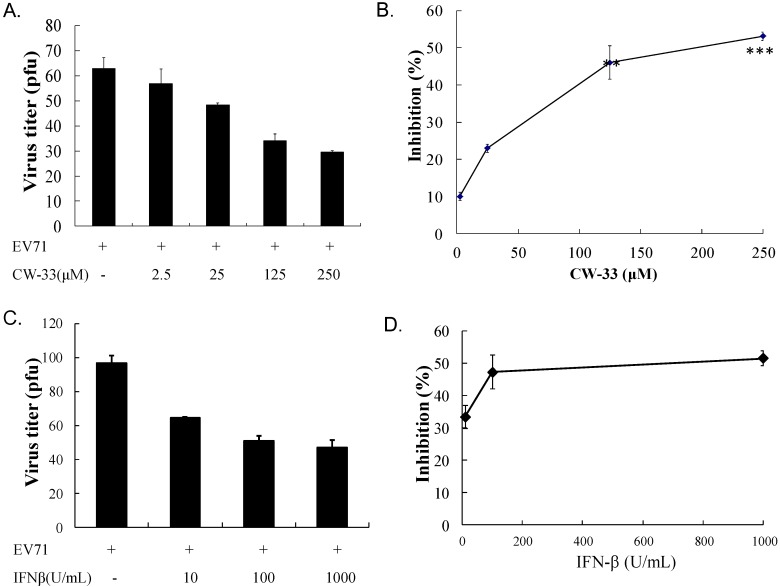
Inhibition of EV-A71 plaque formation by CW-33 or IFN-β alone. Monolayer of RD cells in 6-well plates were infected with EV-A71 (100 pfu), and then immediately treated with indicated concentrations of CW-33 (**A**) or IFN-β (**C**). After 1-h absorption, cell monolayer was washed with PBS, then overlaid with medium containing 1.5% agar. After 48-h incubation, plaque number was counted after staining by 0.1% Crystal Violet, Inhibitory activities of CW-33 (**B**) or IFN-β (**D**) were calculated from ratio of experimental data to mock control. *** *p* value < 0.001 by Scheffe test.

Cytotoxicity of CW-33 to RD cells was initially assessed using MTT assay (Figure 2A). Survival rate exceeded 50% when cells treated with high concentration of CW-33 at 1000 μM, proving CW-33 definitely less cytotoxic. Antiviral activity of CW-33 against EV-A71 was later tested by cytopathic effect inhibition and plaque reduction assay (Figure 2B,C and Figure 3A,B). CW-33 concentration-dependently suppressed EV-A71-induced cytopathic effect in RD cells (Figure 2B), as well as reducing apoptotic rate of virus-infected cells (Figure 2C) (*p* < 0.001). CW-33 likewise showed plaque reduction activity with IC_50_ of 193.8 μM. The results indicated CW-33 displaying a moderate antiviral activity against EV-A71.

### 3.2. Synergistic Antiviral Activity of CW-33 in Combination with Interferon (IFN)-β

**Figure 4:**
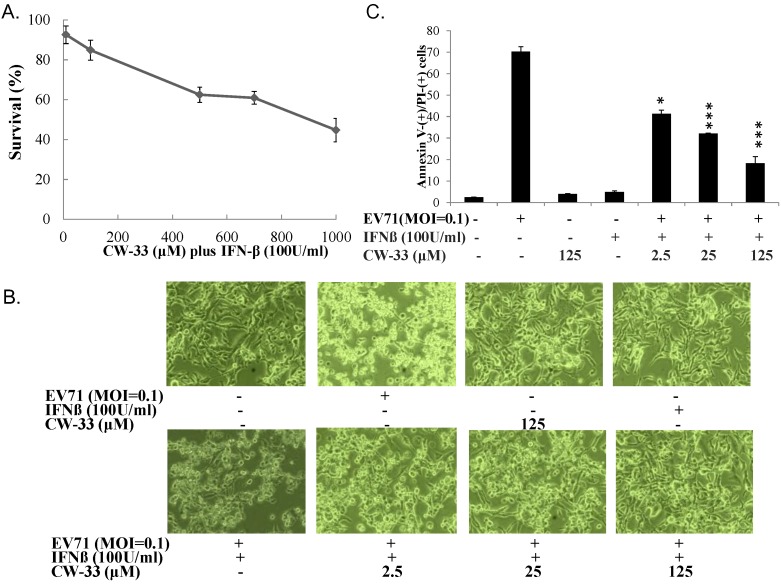
Inhibitory effect of CW-33 in combination with IFN-β on EV-A71-infected cytopathic effect. Firstly, cytotoxicity of CW-33 in combination with IFN-β to RD cells was performed using MTT assays (**A**). RD cells infected with EV-A71 were forthwith treated with CW-33 alone and in combination with IFN-β. Virus-induced cytopathicity was photographed post-infection by phase-contrast microscopy (**B**), apoptosis of infected and/or treated cells analyzed by flow cytometry with Annexin-V/PI staining (**C**). * *p* value < 0.05; *** *p* value < 0.001 by Scheffe test.

**Figure 5:**
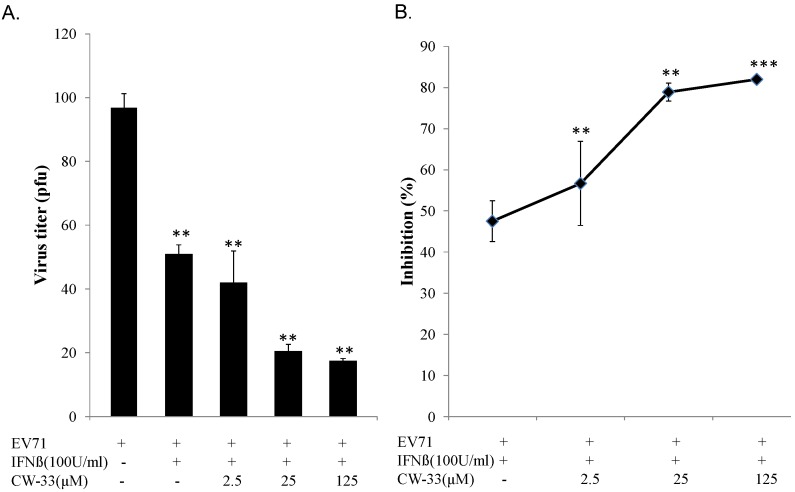
Inhibition of EV-A71 plaque formation by combined treatment of CW-33 with IFN-β. Monolayer of RD cells in 6-well plates infected with EV-A71 (100 pfu) was immediately treated with CW-33 alone or in combination with IFN-β. After 1-h absorption, cell monolayer was washed with PBS, then overlaid with medium containing 1.5% agar. After 48-hour incubation, plaque number was counted after staining by 0.1% Crystal Violet (**A**), 50% inhibitory concentration (IC_50_) calculated from ratio of experimental data to mock control (**B**). ** *p* value < 0.01; *** *p* value < 0.001 by Scheffe test.

**Figure 6:**
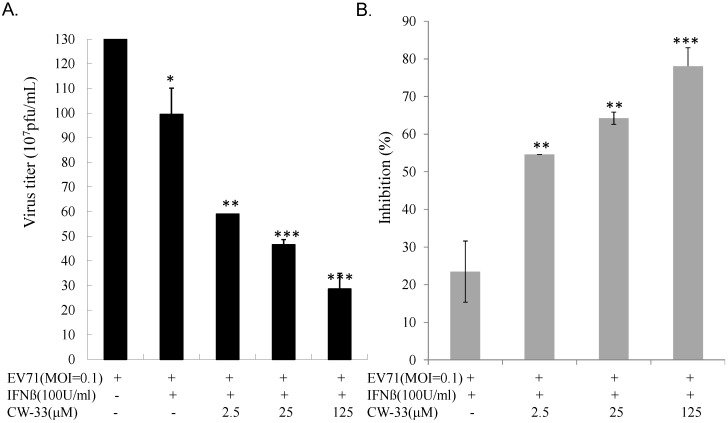
Inhibition of supernatant EV-A71 yield by combined treatment of CW-33 and IFN-β. Cells infected with EV-A71 were immediately treated with CW-33 alone or in combination with IFN-β. Supernatant was harvested 48 h post-infection, virus yield measured by plaque assay (**A**), inhibitory ratio calculated from ratio of experimental data to mock control (**B**). * *p* value < 0.05; ** *p* value < 0.01; *** *p* value < 0.001 by Scheffe test.

IFN-β at a low concentration (100 U/mL) slightly inhibited EV-A71-induced cytopathy (Figure 3C,D). Plaque reduction assay indicated IFN-β exhibiting less antiviral activity (IC_50_ = 966.2 U/mL) against EV-A71 infection. Combined treatment of serial concentrations of CW-33 with 100 U/mL IFN-β showed a more potent inhibition of virus-induced cytopathic effect and apoptosis compared to CW-33 or IFN-β alone (Figure 4 *vs.* Figure 2). For determining additive or synergistic antiviral activity of CW-33 in combination with IFN-β, combined treatment of serial concentrations of CW-33 with 100 U/mL IFN-β was evaluated by EV-A71 plaque and yield reduction assays (Figure 5 and Figure 6). Combined treatment of CW-33 with 100 U/mL IFN-β exhibited synergistic antiviral activities against EV-A71 (IC_50_ of 0.9 μM for plaque reduction and IC_50_ of 1.4 μM for virus yield reduction). CW-33 in combination with 100 U/mL IFN-β exceeded 100-fold lower IC_50_ values against EV-A71 replication *in vitro* compared to CW-33 alone. Results demonstrated a synergistic antiviral activity of CW-33 in combination with a low concentration of IFN-β against EV-A71.

### 3.3. Inhibition of EV-A71 2A Protease by CW-33

**Figure 7:**
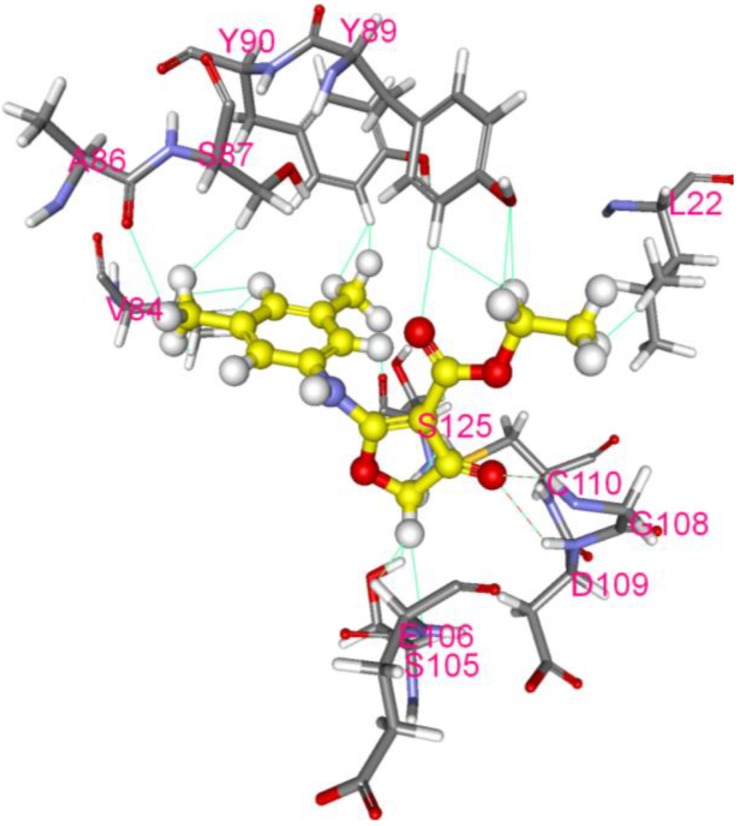
Molecular modeling of interaction between CW-33 and 2A protease. Compound CW-33 (ball and stick, yellow) docked into the active site of EV-A71 2A protease sandwiched between N- and C-terminal domains. Compound CW-33 docked well with 3w95 via hydrogen bonding and hydrophobic reaction, binding amino acids shown as sticks and labeled.

**Figure 8:**
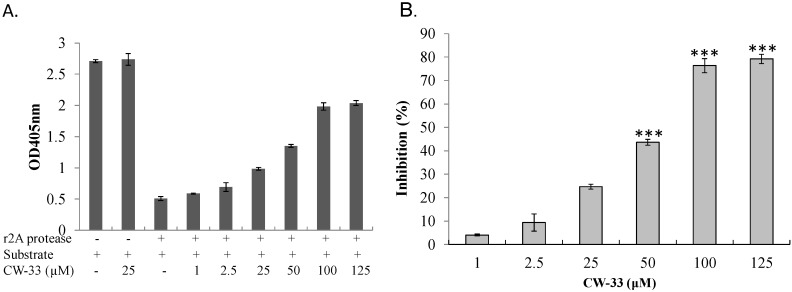
Inhibition by CW-33 on EV-A71 mediated cleavage of 2A protease-specific substrates. For *in vitro* inhibitory enzymatic assay of r2A protease (**A**,**B**), purified r2A protease at 5 µg/mL were added to substrate (10 µg/mL) and forthwith mixed with indicated concentrations of CW-33 for 2 h at 37 °C. Mixtures developed with ABTS/H_2_O_2_ were measured at OD_405_, percentage inhibition of r2A protease activity calculated. *** *p* value < 0.001 by Scheffe test.

With EV-A71 2A protease inhibiting Type I IFN response [11,27], interaction of CW-33 with 2A protease was predicted and analyzed by molecular docking. After global energy optimization, CW-33 was docked into active site of 2A protease consisting of Asn19, His21, Asp39, Tyr90, Gly108, Asp109, Cys110, and Ser125. Modeling of CW-33 and 2A protease had a LibDockScore of 99.6683. Figure 7 showed CW-33 interacting with 2A protease through hydrogen bonding to Gly108 and Asp109 as well as Van der Waals forming among Leu22, Val84, Ala86, Ser87, Tyr89, Tyr90, Ser105, Glu106, Gly108, Asp109, Cys110, and Ser125. These modeling interactions implied that CW-33 bound well to the active site of EV-71A 2A proteinase. To confirm the specific interaction between CW-33 and EV-A71 2A protease, inhibitory effect of CW-33 on the enzymatic activity of 2A protease was tested by *in vitro* cleavage assay with recombinant 2A (r2A) protease (Figure 8). *In vitro* cleavage assay indicated EV-A71 r2A protease significantly cleaving the substrate. Yet, CW-33 exhibited a concentration-dependent relationship with an increase of remaining substrate, revealing dose-dependent inhibition of r2A protease activity with IC_50_ of 53.1 μM (Figure 8A,B). *In vitro* cleavage of r2A protease assays revealed CW-33 manifesting an inhibitory effect on EV-A71 2A protease activity in dose-dependent manners. Results confirmed CW-33 specifically binding to the active site of EV-A71 2A protease.

### 3.4. Recovery of IFN-Stimulated Tyk2/ STAT1 Signaling in Infected Cells by CW-33

**Figure 9:**
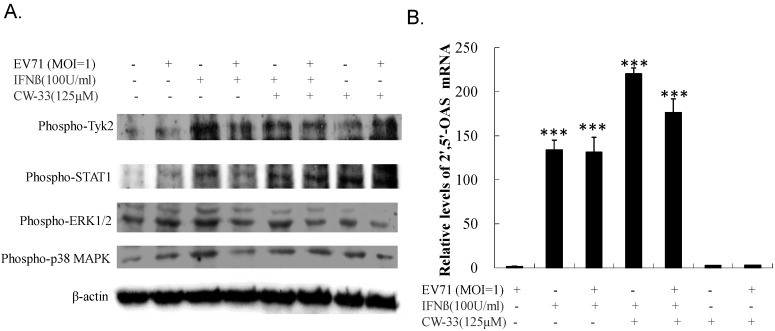
Activation of Tyk2/STAT1 signaling in EV-A71 infected cells by combined treatment of CW-33 and IFN-β. For assessing phosphorylation of Tyk2 and STAT1 (**A**), lysates of infected cells treated with or without CW-33, IFN-β, or combination thereof were resolved on 10% SDS-PAGE and transferred onto nitrocellulose paper. Blot was probed with specific mAbs, developed with enhanced chemiluminescence substrates. Relative mRNA expression of Tyk2/STAT1-dependent gene 2′-5′ OAS was measured with Real-time RT-PCR 36 h post infection and treatment, and then normalized by housekeeping gene GAPDH (**B**). *** *p* value < 0.001 by Scheffe test.

Western blot of Tyk2, STAT1, ERK1/2, and p38 MAPK phosphorylation used lysate from (un)infected cells treated with or without CW-33 alone and in combination with IFN-β. Figure 9A shows IFN-β strongly inducing phosphorylation of Tyk2, STAT1, ERK1/2, and p38 MAPK (Lane 3); EV-A71 repressed IFN-β-induced phosphorylation (Lane 4). Interestingly, CW-33 restored IFN-β-stimulated phosphorylation of Tyk2 and STAT1, but not ERK1/2 and p38 MAPK in infected cells compared to those in infected cells treated with IFN-β alone (Lane 6 *vs.* Lane 4). For examining the activation of STAT1-mediated genes, the mRNA expression of IFN-stimulated gene 2′,5′-OAS was quantified by real-time PCR (Figure 9B). IFN-β alone stimulated up-regulation of 2′,5′-OAS in uninfected cells; EV-A71 infection had no effect on 2′,5′-OAS mRNA level in response to IFN-β. Combination of CW-33 and IFN-β triggered a higher level of 2′,5′-OAS mRNA than IFN-β alone in infected cells. Both Western blot and quantitative PCR indicated CW-33 reducing the antagonistic effect of EV-A71 on Type I IFN signaling pathway.

### 3.5. Inhibitory Effect on Viral 2A Protease-Mediated Cleavage of IFNAR1

**Figure 10:**
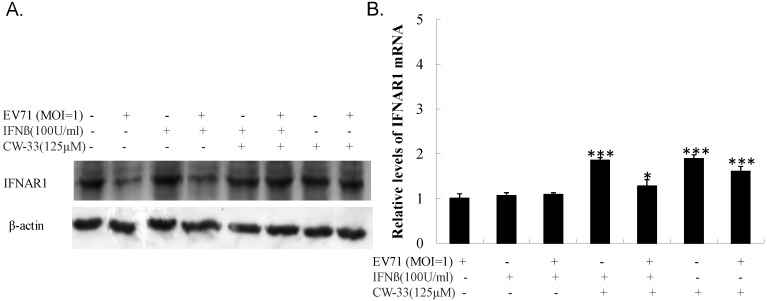
Recovery of IFNAR1 protein levels in EV-A71-infected cells by combined treatment of CW-33 and IFN-β. For analyzing protein levels of IFNAR1 (**A**), lysates of infected cells treated with or without CW-33, IFN-β, or combination were separated by 10% SDS-PAGE and transferred onto nitrocellulose paper. Blot was probed with specific mAb against IFNAR1, developed with enhanced chemiluminescence substrates. Cells were harvested for measuring IFNAR1 mRNA expression using Real-time RT-PCR36 h post-infection and treatment (**B**). * *p* value < 0.05; *** *p* value < 0.001 by Scheffe test.

Antagonistic effect of EV-A71 on type I IFN signaling demonstrably correlated with the cleavage of Type IFN receptor 1 (IFNAR1) by 2A protease. Western blot indicated lower IFNAR1 levels in infected *versus* un-infected cells (Figure 10A, Lane 2 *vs.* Lane 1). IFN-β treatment did not restore IFNAR1 level in infected cells (Figure 10A, Lane 4), yet CW-33 alone and in combination with IFN-β significantly recovered the protein level of IFNAR1 in infected cells (Figure 10A, Lanes 6 and 8). Real-time PCR indicated no significant change of IFNAR1 mRNA in infected cells treated with CW-33 alone and in combination with IFN-β (Figure 10B). Results highlighted CW-33 suppressing the cleavage action of EV-A71 2A protease on IFNAR1, elucidating the synergistic mechanism of CW-33 in combination with IFN-β on activation of Tyk2/STAT1 signaling pathway and induction of IFN-stimulated genes in EV-A71 infected cells.

## 4. Discussion

This study showed that IFN-β was less effective against EV-A71 (Figure 3C,D), consistent with prior reports in which EV-A71 antagonized antiviral actions of Type I IFN [10,27,29]. EV-A71 2A protease cleaved IFN receptor 1, reducing IFN-mediated activation of Jak1, Tyk2, STAT1, and STAT2, interfering with Type I IFN signal. EV-A71 2A protease specifically sliced mitochondrial antiviral signaling (MAVS) protein, inactivating antiviral innate immune response of retinoic acid induced gene-I (RIG-I) and melanoma differentiation associated gene (MDA-5), lowering the production of Type I IFN. Prior reports cited the pivotal role of 2A protease in Type I IFN antagonism of EV-A71.

While CW-33 exhibited a moderate activity against EV-A71 (IC_50_ = 171.2 μM for plaque reduction) (Figure 2 and Figure 3), *in vitro* cleavage of r2A protease assays indicated CW-33 alone showing specific inhibition with an IC50 of 53.1 μM on enzymatic activity of viral 2A protease (Figure 8A,B). This asserts modeled interaction of CW-33 with the active site of EV-A71 2A protease (Figure 7). CW-33 fully reduced EV-A71-induced apoptosis, restraining the cleavage of IFNAR1 in infected cells (Figure 2B and Figure 10A). CW-33, specifically binding to viral 2A protease, attenuated Type I IFN antagonism of EV-A71 via blocking 2A protease-mediated cleavage of IFNAR1 (Figure 10). CW-33 plus IFN-β manifested a synergistic inhibition of EV-A71 replication *in vitro*: e.g., cytopathic repression, plaque reduction, and virus yield decrease (Figure 4, Figure 5 and Figure 6). Combination of Type I IFN and antiviral drugs (ribavirin, boceprevir, and telaprevir) has seen clinical use in treating hepatitis B and C [7,8]. This study verified the synergistic activity of CW-33 and IFN-β against EV-A71. Low concentration of IFN-β (100 U/mL) in combination with CW-33 exhibited therapeutic potential against EV-A71, easing clinical side-effects of Type I IFNs at high dose. Combination of 2A protease-specific inhibitors and Type I IFNs could exhibit the synergistic antiviral activity, opening up a novel approach for formulating effective antiviral agents against EV-A71.

## Abbreviations

EV-A71, Enterovirus A71; IFN, interferon; r2A, recombinant 2A protease; CW-33, ethyl 2-(3′,5′-dimethylanilino)-4-oxo-4,5-dihydrofuran-3-carboxylate; CVA, Coxsackie A virus; CVB, Coxsackie B virus; IFNAR1, IFN-α/β receptor 1; eIF4G, eukaryotic translation initiation factor 4G; IC50, 50% inhibitory concentration.

## References

[1] Lin T.Y., Twu S.J., Ho M.S., Chang Y.L., Lee C.Y. (2003). Enterovirus 71 outbreaks, Taiwan: Occurrence and recognition. Emerg. Infect. Dis..

[2] Melnick J.L. (1993). The discovery of the enteroviruses and the classification of poliovirus among them. Biologicals.

[3] Mettenleiter T.C., Sobrino F. (2008). Foot-and-Mouth Disease Virus. Animal Viruses: Molecular Biology.

[4] Oberste M.S., Maher K., Kilpatrick D.R., Flemister M.R., Brown B.A., Pallansch M.A. (1999). Typing of human enteroviruses by partial sequencing of VP1. J. Clin. Microbiol..

[5] Wang J.R., Tuan Y.C., Tsai H.P., Yan J.J., Liu C.C., Su I.J. (2002). Change of major genotype of enterovirus 71 in outbreaks of hand-foot-and-mouth disease in Taiwan between 1998 and 2000. J. Clin. Microbiol..

[6] Shang L., Xu M., Yin Z. (2013). Antiviral drug discovery for the treatment of enterovirus 71 infections. Antivir. Res..

[7] Cooksley W.G. (2004). The role of interferon therapy in hepatitis B. Med. Gen. Med..

[8] Shepherd J., Waugh N., Hewitson P. (2000). Combination therapy (interferon alfa and ribavirin) in the treatment of chronic hepatitis C: A rapid and systematic review. Health Technol. Assess..

[9] Li Z.H., Li C.M., Ling P., Shen F.H., Chen S.H., Liu C.C., Yu C.K., Chen S.H. (2008). Ribavirin reduces mortality in enterovirus 71-infected mice by decreasing viral replication. J. Infect. Dis..

[10] Liu M.L., Lee Y.P., Wang Y.F., Lei H.Y., Liu C.C., Wang S.M., Su I.J., Wang J.R., Yeh T.M., Chen S.H. (2005). Type I interferons protect mice against enterovirus 71 infection. J. Gen. Virol..

[11] Lu J., Yi L., Zhao J., Yu J., Chen Y., Lin M.C., Kung H.F., He M.L. (2012). Enterovirus 71 disrupts interferon signaling by reducing the level of interferon receptor 1. J. Virol..

[12] Zhang G., Zhou F., Gu B., Dong C., Feng D., Xie F., Wang J., Zhang C., Cao Q., Deng Y. (2012). *In vitro* and *in vivo* evaluation of ribavirin and pleconaril antiviral activity against enterovirus 71 infection. Arch. Virol..

[13] Dragovich P.S., Prins T.J., Zhou R., Webber S.E., Marakovits J.T., Fuhrman S.A., Patick A.K., Matthews D.A., Lee C.A., Ford C.E. (1999). Structure-based design, synthesis, and biological evaluation of irreversible human rhinovirus 3C protease inhibitors. 4. Incorporation of P1 lactam moieties as L-glutamine replacements. J. Med. Chem..

[14] Zhang X.N., Song Z.G., Jiang T., Shi B.S., Hu Y.W., Yuan Z.H. (2010). Rupintrivir is a promising candidate for treating severe cases of Enterovirus-71 infection. World J. Gastroenterol..

[15] Shia K.S., Shih S.R., Chang C.M. (2004). Imidazolidinone Compounds. US Patant.

[16] Cui S., Wang J., Fan T., Qin B., Guo L., Lei X., Wang M., Jin Q. (2011). Crystal structure of human enterovirus 71 3C protease. J. Mol. Biol..

[17] Hung H.C., Wang H.C., Shih S.R., Teng I.F., Tseng C.P., Hsu J.T. (2011). Synergistic inhibition of enterovirus 71 replication by interferon and rupintrivir. J. Infect. Dis..

[18] Tarus P.K., Coombes P.H., Crouch N.R., Mulholland D.A., Moodley B. (2005). Furoquinoline alkaloids from the southern African Rutaceae Teclea natalensis. Phytochemistry.

[19] Zhao W., Wolfender J.L., Hostettmann K., Xu R., Qin G. (1998). Antifungal alkaloids and limonoid derivatives from Dictamnus dasycarpus. Phytochemistry.

[20] Severino V.G., da Silva M.F., Lucarini R., Montanari L.B., Cunha W.R., Vinholis A.H., Martins C.H. (2009). Determination of the antibacterial activity of crude extracts and compounds isolated from *Hortia oreadica* (Rutaceae) against oral pathogens. Braz. J. Microbiol..

[21] Kiplimo J.J., Islam M.S., Koorbanally N.A. (2011). A novel flavonoid and furoquinoline alkaloids from Vepris glomerata and their antioxidant activity. Nat. Prod. Commun..

[22] Wansi J.D., Mesaik M.A., Chiozem D.D., Devkota K.P., Gaboriaud-Kolar N., Lallemand M.C., Wandji J., Choudhary M.I., Sewald N. (2008). Oxidative burst inhibitory and cytotoxic indoloquinazoline and furoquinoline alkaloids from Oricia suaveolens. J. Nat. Prod..

[23] Kuo S.C., Huang S.C., Hung L.J., Cheng H.E., Lin T.P., Wu C.H., Ishii K., Nakamura H. (1991). Studies of heterocyclic compounds. VIII. Synthesis, anti-inflammatory and antiallergic activities of *N*-alkyl-2,3,4,9-tetrahydrofuro[2,3-b]quinoline-3,4-diones and related compounds. J. Heterocycl. Chem..

[24] Su M.J., Chang G.J., Wu M.H., Kuo S.C. (1997). Electrophysiological basis for the antiarrhythmic action and positive inotropy of HA-7, a furoquinoline alkaloid derivative, in rat heart. Br. J. Pharmacol..

[25] Lan Y.C., Lin T.H., Tsai J.D., Yang Y.C., Peng C.T., Shih M.C., Lin Y.J., Lin C.W. (2011). Molecular epidemiology of the 2005 enterovirus 71 outbreak in central Taiwan. Scand. J. Infect. Dis..

[26] Wang C.Y., Huang S.C., Zhang Y., Lai Z.R., Kung S.H., Chang Y.S., Lin C.W. (2012). Antiviral ability of *Kalanchoe gracilis* leaf extract against Enterovirus 71 and Coxsackievirus A16. Evid. Based Complement. Alternat. Med..

[27] Wang C.Y., Huang S.C., Lai Z.R., Ho Y.L., Jou Y.J., Kung S.H., Zhang Y., Chang Y.S., Lin C.W. (2013). Eupafolin and Ethyl acetate fraction of *Kalanchoe gracilis* stem extract show potent antiviral activities against Enterovirus 71 and Coxsackievirus A16. Evid. Based Complement. Alternat. Med..

[28] Mu Z., Wang B., Zhang X., Gao X., Qin B., Zhao Z., Cui S. (2013). Crystal structure of 2A proteinase from hand, foot and mouth disease virus. J. Mol. Biol..

[29] Yi L., He Y., Chen Y., Kung H.F., He M.L. (2011). Potent inhibition of human enterovirus 71replication by type I interferon subtypes. Antivir. Ther..

